# Dapsone Toxicity in Relapsing Polychondritis: Methemoglobinemia and Concomitant Hemolytic Anemia

**DOI:** 10.7759/cureus.101910

**Published:** 2026-01-20

**Authors:** José Nuno Magalhães, Ana Monteiro

**Affiliations:** 1 Medicina, Unidade Local de Saúde de Santo António, Porto, PRT; 2 Internal Medicine, Unidade Local de Saúde de Santo António, Porto, PRT

**Keywords:** dapsone toxicity, drug-induced hemolytic anemia, methemoglobinemia, relapsing polychondritis, saturation gap

## Abstract

Relapsing polychondritis (RP) is a rare immune-mediated disorder characterized by episodic inflammation of cartilage and other proteoglycan-rich tissues. Dapsone is sometimes used as a steroid-sparing agent in selected phenotypes, but its oxidative metabolites can cause clinically significant methemoglobinemia and hemolysis. We describe a 70-year-old man with auricular chondritis and biopsy-supported RP who developed progressive dyspnea and cyanosis after three months of dapsone 100 mg daily. Oxygen saturation remained low despite supplemental oxygen, while arterial oxygen tension was preserved, prompting co-oximetry that confirmed methemoglobinemia. He improved rapidly after methylene blue administration, and dapsone discontinuation prevented recurrence. During the same admission, anemia worsened with laboratory and smear features consistent with oxidant hemolysis and a negative direct antiglobulin test, improving after drug withdrawal. This case highlights the diagnostic value of the "saturation gap" and the need to actively consider dapsone-related dyshemoglobinemia and oxidant hemolysis in patients with refractory low pulse oximetry readings or worsening anemia while on therapy, enabling timely confirmation and prompt drug withdrawal.

## Introduction

Relapsing polychondritis (RP) is an immune-mediated, episodic inflammatory disorder targeting cartilage and other proteoglycan-rich tissues, with potentially progressive deformity and, at the severe end of the spectrum, organ dysfunction [1]. Clinical expression spans self-limited auricular or nasal chondritis to severe airway and cardiovascular involvement, and this heterogeneous presentation often leads to diagnostic delay [1,2]. Owing to its rarity, high-quality comparative data are scarce, and, in the absence of randomized trials, treatment is largely empirical and tailored to the dominant organ manifestations and disease severity [3]. Dapsone is a sulfone with antimicrobial and neutrophil-modulating anti-inflammatory activity that has been used as a steroid-sparing agent across several neutrophilic and autoimmune dermatoses [4]. In RP, dapsone has been reported as a therapeutic option in selected patients, based mainly on early case series and individual reports [1,3]. Because dapsone’s hydroxylamine metabolites predictably oxidize hemoglobin and disrupt erythrocyte membrane integrity, hematologic toxicity can occur during therapy. This may manifest as methemoglobinemia and hemolysis, and clinically significant cases may be missed without a high index of suspicion [4,5]. Here, we report a case of dapsone-associated methemoglobinemia with concomitant hemolytic anemia in a patient with RP, underscoring the need for prompt recognition and drug withdrawal when refractory low oxygen saturation and anemia develop during therapy [5-7].

## Case presentation

**Figure 1:**
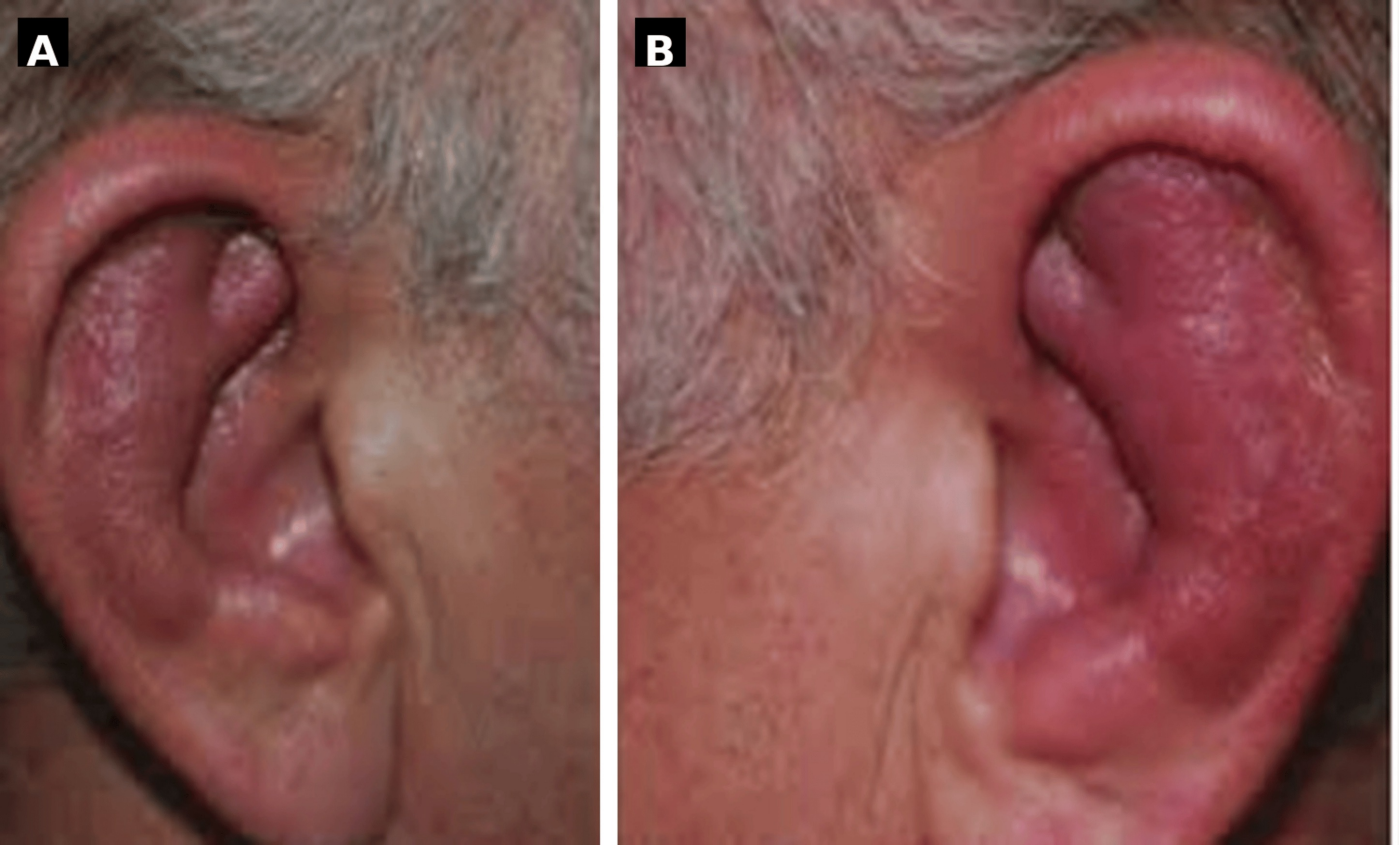
Bilateral auricular chondritis in relapsing polychondritis. (A–B) Clinical photographs showing erythema and swelling of the auricular cartilage with relative lobule sparing, consistent with bilateral auricular chondritis.

A 70-year-old man with a personal history of type 2 diabetes mellitus, chronic obstructive pulmonary disease, and chronic anemia presented to the emergency department on multiple occasions with persistent auricular inflammation that had not responded to prior oral nonsteroidal anti-inflammatory drugs (NSAIDs) and antibiotics. Physical examination revealed isolated auricular chondritis (Figure 1), and laboratory testing showed a mild elevation of inflammatory markers.

Given the persistence of symptoms, a biopsy was performed; histopathology was compatible with relapsing polychondritis and, together with the clinical presentation, supported the diagnosis. Because long-term systemic glucocorticoids were considered undesirable given his comorbid diabetes, a steroid-sparing strategy was pursued. After glucose-6-phosphate dehydrogenase deficiency was excluded, dapsone was initiated at 100 mg/day, with subsequent improvement in auricular inflammation.

After approximately three months of treatment, he was admitted with progressive asthenia and worsening dyspnea. On examination, he was tachypneic, with peripheral cyanosis and low oxygen saturation on pulse oximetry (80% on room air), which failed to respond to supplemental oxygen. Arterial blood gas analysis showed preserved arterial oxygen tension despite persistently low oxygen saturation, raising suspicion for dyshemoglobinemia; co-oximetry confirmed methemoglobinemia at 15% (Table 1).

**Table 1:** Arterial blood gas and co-oximetry on admission, demonstrating a saturation gap with methemoglobinemia. PaO₂, arterial partial pressure of oxygen; PaCO₂, arterial partial pressure of carbon dioxide

Parameter	Result	Units	Normal range
pH	7.41		7.35-7.45
PaO_2_	75.1	mmHg	75.0-100.0
PaCO_2_	38.5	mmHg	35.0-48-0
Oxyhemoglobin	79.9	%	94.0-97.0
Carboxyhemoglobin	0.5	%	0.5-1.5
Methemoglobin	15.0	%	0.0-1.5

Methylene blue was administered intravenously at a dose of 2 mg/kg, resulting in a rapid decline in the methemoglobin fraction to 0.7% over the following hours, with parallel improvement in dyspnea and peripheral oxygen saturation. Dapsone was discontinued, and methemoglobinemia did not recur after drug withdrawal.

During the same hospitalization, his anemia worsened from baseline, and an extended laboratory evaluation for hemolysis was performed (Table 2), showing marked reticulocytosis, elevated lactate dehydrogenase, low haptoglobin, and negative direct and indirect antiglobulin tests. Peripheral blood smear demonstrated bite cells consistent with oxidant injury.

**Table 2:** Laboratory evaluation of worsening anemia during dapsone therapy, supporting non-immune hemolysis. Baseline chronic hemoglobin was 9 g/dL prior to dapsone initiation.

Parameter	Result	Units	Normal range
Erythrocytes	2.38	x10^6^/µL	4.5-5.5
Hemoglobin	6.3	g/dL	13-17
Hematocrit	19.4	%	40.0-50.0
Platelets	311.0	x10^3^/µL	150.0-400.0
Reticulocytes (absolute value)	280.01	x10^9^/L	50.0-100.0
Reticulocytes (percentage)	8.26	%	0.5-2.5
Sedimentation rate (1st hour)	43	mm	0-20
Total bilirubin	1.03	mg/dL	0.2.-1.00
Direct bilirubin	0.55	mg/dL	0.00-0.30
Lactate dehydrogenase	348.0	U/L	135.0-225.0
Reactive C Protein	39.6	mg/L	0.0-5.0
Haptoglobin	< 0.24	g/L	0.45-2.05
Glucose-6-phosphate dehydrogenase	15.8	U/g Hb	7.0-20.4
Direct antiglobulin test	Negative		
Indirect antiglobulin test	Negative		

Given suspected dapsone-related toxicity, therapy for relapsing polychondritis was transitioned to low-dose oral corticosteroids, with favorable clinical and laboratory evolution and no reported clinical relapse at three-month follow-up.

## Discussion

RP remains a rare, clinically heterogeneous immune-mediated disorder for which therapeutic decisions are frequently individualized to organ involvement and overall risk profile [2,3]. Within that landscape, dapsone has historically been used as a steroid-sparing option in selected, usually less severe RP phenotypes; nevertheless, its dose-related oxidative hematologic toxicity is well recognized and should be regarded as an anticipated constraint requiring active surveillance [3-5]. Clinically significant toxicity with methemoglobinemia and hemolytic anemia can occur at therapeutic doses, even when baseline glucose-6-phosphate dehydrogenase deficiency screening is negative, reflecting that normal enzymatic activity reduces but does not abolish oxidant vulnerability [5]. In addition, dapsone displays a relatively prolonged half-life and enterohepatic recirculation, which can contribute to persistence or rebound of methemoglobinemia after initial reversal, even after treatment is instituted [5,8].

In our patient, methemoglobinemia was the dominant acute presentation. In this setting, the decisive diagnostic signal is the “saturation gap”: cyanosis and low pulse oximetry readings that do not meaningfully correct with supplemental oxygen despite a preserved arterial oxygen tension [6,7]; confirmation requires co-oximetry, which directly quantifies methemoglobin and resolves the discordance between pulse oximetry and arterial oxygen tension [7]. In this case, a methemoglobin fraction of 15% with rapid clinical and analytical response to methylene blue supported the diagnosis of acquired, drug-mediated methemoglobinemia and strengthened the attribution to dapsone. Management is centered on immediate discontinuation of the oxidant drug, with intravenous methylene blue - commonly 1-2 mg/kg - reserved for symptomatic patients or clinically significant methemoglobin elevations [6]. Given dapsone pharmacokinetics, post-treatment monitoring for rebound is clinically relevant, and selected cases may require repeat methylene blue dosing [5,8]. Importantly, laboratory assessments of oxygenation in methemoglobinemia show that pulse oximetry may plateau around 85% across a range of methemoglobin fractions, consistent with the refractory low pulse oximetry readings observed in our patient and reinforcing the bedside diagnostic value of recognizing this discordance [7]. Similarly, published case reports and clinical reviews of dapsone-induced methemoglobinemia describe clinically meaningful dyshemoglobinemia at standard therapeutic dosing with prompt improvement after methylene blue and drug withdrawal, consistent with our patient's course [5]. This presentation has also been reported in RP patients treated with dapsone as part of a steroid-sparing strategy [9], and our case adds to this by emphasizing the bedside diagnostic pivot of the “saturation gap,” leading to prompt co-oximetry confirmation and treatment.

The clinical scenario also included hemolytic anemia, the other major expression of dapsone hemotoxicity. Dapsone's hydroxylamine metabolites impose oxidative stress on erythrocytes, leading to membrane injury and characteristic smear findings, such as bite cells, alongside laboratory evidence of hemolysis [4,5]. In our patient, worsening anemia with a hyperproliferative response, low haptoglobin, elevated lactate dehydrogenase, bite cells, and a negative direct antiglobulin test - followed by improvement after dapsone discontinuation - supports dapsone-related oxidant hemolysis as the most parsimonious explanation for the hematologic deterioration observed under therapy [10]. While dapsone-associated hemolytic anemia has also been described in RP [11], our case is distinctive in documenting clinically significant methemoglobinemia and oxidant hemolysis during the same admission, supporting a low threshold to evaluate concurrently for both complications when refractory low pulse oximetry, cyanosis, or worsening anemia develops under dapsone in RP.

## Conclusions

Dapsone may be considered a steroid-sparing option in selected relapsing polychondritis phenotypes, but its use is constrained by dose-related oxidative hematologic toxicity. In patients who develop cyanosis or refractory low pulse oximetry readings with preserved arterial oxygen tension, methemoglobinemia should be promptly suspected and confirmed by co-oximetry. Even with normal baseline glucose-6-phosphate dehydrogenase activity, concomitant hemolytic anemia can occur; drug withdrawal is the key intervention for both toxicities, while methylene blue is reserved for clinically significant methemoglobinemia in appropriate patients.
